# Functional Genomic Screening Independently Identifies CUL3 as a Mediator of Vemurafenib Resistance via Src-Rac1 Signaling Axis

**DOI:** 10.3389/fonc.2020.00442

**Published:** 2020-04-03

**Authors:** Marion Vanneste, Charlotte R. Feddersen, Afshin Varzavand, Elliot Y. Zhu, Tyler Foley, Lei Zhao, Kathleen H. Holt, Mohammed Milhem, Robert Piper, Christopher S. Stipp, Adam J. Dupuy, Michael D. Henry

**Affiliations:** ^1^Department of Molecular Physiology and Biophysics, Roy J. and Lucille A. Carver College of Medicine, The University of Iowa, Iowa City, IA, United States; ^2^Department of Anatomy and Cell Biology, Carver College of Medicine, University of Iowa, Iowa City, IA, United States; ^3^Department of Biology, College of Liberal Arts and Sciences, University of Iowa, Iowa City, IA, United States; ^4^Carver College of Medicine, University of Iowa, Iowa City, IA, United States; ^5^Viral Vector Core Facility, Carver College of Medicine, University of Iowa, Iowa City, IA, United States; ^6^Department of Internal Medicine, Carver College of Medicine, University of Iowa, Iowa City, IA, United States; ^7^Holden Comprehensive Cancer Center, University of Iowa, Iowa City, IA, United States; ^8^Department of Radiation Oncology, Roy J. and Lucille A. Carver College of Medicine, The University of Iowa, Iowa City, IA, United States; ^9^Department of Pathology, Roy J. and Lucille A. Carver College of Medicine, The University of Iowa, Iowa City, IA, United States; ^10^Department of Urology, Roy J. and Lucille A. Carver College of Medicine, The University of Iowa, Iowa City, IA, United States

**Keywords:** melanoma, forward genetic screen, MAPKi resistance, CUL3 ubiquitin ligase, Rac1, Src inhibitor

## Abstract

Patients with malignant melanoma have a 5-year survival rate of only 15–20% once the tumor has metastasized to distant tissues. While MAP kinase pathway inhibitors (MAPKi) are initially effective for the majority of patients with melanoma harboring BRAF^V600E^ mutation, over 90% of patients relapse within 2 years. Thus, there is a critical need for understanding MAPKi resistance mechanisms. In this manuscript, we performed a forward genetic screen using a whole genome shRNA library to identify negative regulators of vemurafenib resistance. We identified loss of NF1 and CUL3 as drivers of vemurafenib resistance. NF1 is a known driver of vemurafenib resistance in melanoma through its action as a negative regulator of RAS. However, the mechanism by which CUL3, a key protein in E3 ubiquitin ligase complexes, is involved in vemurafenib resistance was unknown. We found that loss of CUL3 was associated with an increase in RAC1 activity and MEK^S298^ phosphorylation. However, the addition of the Src family inhibitor saracatinib prevented resistance to vemurafenib in CUL3^KD^ cells and reversed RAC1 activation. This finding suggests that inhibition of the Src family suppresses MAPKi resistance in CUL3^KD^ cells by inactivation of RAC1. Our results also indicated that the loss of CUL3 does not promote the activation of RAC1 through stabilization, suggesting that CUL3 is involved in the stability of upstream regulators of RAC1. Collectively, our study identifies the loss of CUL3 as a driver of MAPKi resistance through activation of RAC1 and demonstrates that inhibition of the Src family can suppress the MAPKi resistance phenotype in CUL3^KD^ cells by inactivating RAC1 protein.

## Background

Melanoma is the deadliest form of skin cancer, causing nearly 10,000 deaths per year (1). While recurrence of stage I and II melanoma can be effectively prevented by wide local excision as the sole therapy (5-year survival rates around 95 and 75%, respectively), metastatic melanoma is more difficult to treat (5-year survival around 56 and 18% for stage III and IV, respectively). Current treatment options include radiation, chemotherapy, targeted therapy, and more recently immunotherapy (2). Fortunately, 63% of late stage melanomas harbor BRAF^V600^ mutations and can be treated with vemurafenib, a highly selective kinase inhibitor that specifically targets the mutant protein (3, 4). However, while vemurafenib initially provides complete or partial response in over 50% of patients, the majority of patients relapse once tumors acquire resistance to vemurafenib (5). The addition of the MEK inhibitor (MEKi) cobimetinib extended progression free survival and overall survival in these patients. However, again, the majority of patients relapse once resistance occurs (6). Therefore, understanding the mechanisms of MAPKi resistance could provide diagnostic information to predict patients' responses to MAPKi therapy and may also identify novel drug combinations that can delay or prevent MAPKi resistance.

Genetic analysis of progression samples has provided key insights into the most common genetic resistance mechanisms, including *BRAF*^*V600*^ mutant amplification, BRAF^V600^ mutant truncations, mutant BRAF^V600^ fusions, RAS genes (*NRAS, KRAS, HRAS*), *RAC1, MAP2K1* or *AKT* gain-of-function mutations, and loss of function events in *CDKN2A, PTEN, PIK3R2*, and *DUSP4* [for review, Kakadia et al. (7)]. However, these mechanisms explain only 60–70% of cases of BRAFi resistance, leaving a substantial number of resistance mechanisms yet to be identified. Moreover, some of these may not be bona fide resistance drivers on their own as, for example, A375 and SK-MEL-28 cells are CDKN2A mutant and PTEN deficient, respectively, and yet sensitive to vemurafenib. Forward genetic screens have been used for years to study important cancer phenotypes and, more recently, these screens have been developed to understand how loss- or gain-of-function events can drive resistance to BRAFi (8–13). The genetic approaches used to investigate BRAFi resistance include libraries of near-genome-wide reagents such as ORFs, shRNA, and CRISPR guides and can further be subdivided into arrayed or pooled screens. In a pooled screen, each element must provide a selective advantage to cells bearing that element compared to the others in the pool and therefore this format better represents the heterogeneous clonal evolution of cancer. Arrayed screens confer a higher sensitivity since each ORF or guide is tested separately for the phenotype. However, these screens require robotic liquid handling and high-throughput cell analysis instruments, preventing many research laboratories from utilizing this approach. They also do not recapitulate the mixed clonal population of cells that are present during cancer development.

Although multiple screens have been performed to identify drivers of BRAFi resistance, there has been little overlap in the genes identified in the respective screens despite using the same A375 human melanoma cell line (8–13). More specifically, when analyzing the identified loss of function drivers from four screens, only NF1 was identified across all four screens and only seven other genes were identified by three studies and included NF2 and CUL3 (8, 12, 13). Most identified genes (35/48) were only identified by one study. This is even more evident when analyzing the identified gain of function drivers from another four screens (9–11, 13). No gene was identified in all four screens, 70/88 genes were only identified in a single screen, and BRAF overexpression—a known mechanism of resistance—was only identified in two of the screens. When these screens are analyzed together, there are certain patterns of resistance that emerge. In terms of loss of function drivers, many members of the E2/E3 ubiquitin ligase complex, the mediator complex, and the STAGA or SAGA complex are implicated in mediating vemurafenib resistance. Gain of function drivers include G-protein coupled receptors such as lysophosphatidic acid receptors (LPAR), kinases including BRAF and RAF, and receptor tyrosine kinases including Src family members Src, BTK, HCK, and LCK. Due to the lack of reproducibility between screens, we wanted to perform a separate shRNA-based screen using a whole-genome shRNA library and compare our results to previous published findings. In doing so, our aim was to discover shared mechanisms of MAPKi resistance across screens with the hope that these shared mechanisms will be more clinically applicable.

Here we used a shRNA library consisting of 113,001 shRNAs covering 18,938 genes to identify negative regulators of resistance to vemurafenib in the BRAF^V600E^-expressing human melanoma cell line A375. We identified loss of NF1 and CUL3, identified in previous screens, as drivers of vemurafenib resistance. Loss of CUL3 was associated with an increase of RAC1 activity and MEK^S298^ phosphorylation. The Src family inhibitor saracatinib decreased both RAC1 activity and MEK^S298^ phosphorylation and reversed resistance to vemurafenib in CUL3^KD^ cells, suggesting that inhibition of the Src family suppresses the MAPKi resistance phenotype in CUL3^KD^ cells by inactivating RAC1 protein. We demonstrated that RAC1-driven mechanisms of resistance to MAPKi could be prevented through Src inhibition, providing new options for future targeted therapies in melanoma.

## Materials and Methods

### Cell Culture and Inhibitors

A375 and 451.Lu parental cell lines were obtained from the ATCC and grown in DMEM (Gibco) supplemented with 10% FBS (Gibco) and 1% NEAA (Gibco). Media for A375 and 451.Lu KD derivatives was also supplemented with puromycin (Gold Biotechnology) 0.5 μg/ml. Vemurafenib and saracatinib (Selleckchem) were used at 3 and 2 μM, respectively.

### Lentiviral shRNA Screen

**Figure 1:**
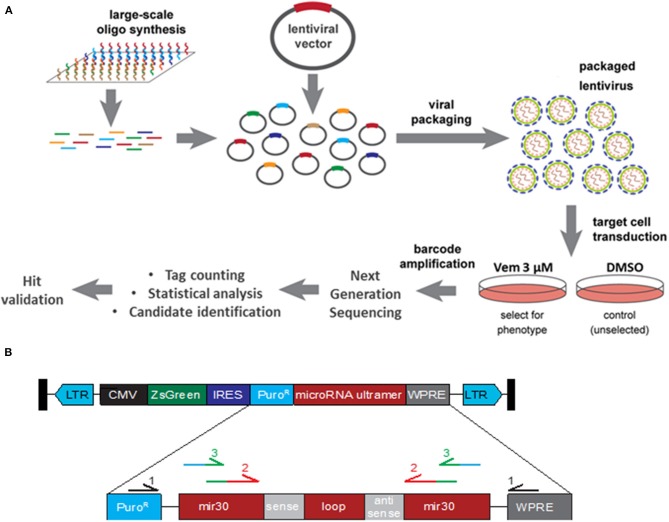
Functional Genomic Screening Overview. **(A)** Functional Genomic Screening Overview. shRNA screening steps include: viral library preparation, target cell infection, survival selection, guide/ORF recovery by amplification, NGS library preparation, NGS, statistical analysis, candidate identification and validation of selected hits. **(B)** shRNA construct design. pZIP lentiviral vector contains a CMV promoter driving expression of a ZsGreen cassette, a puromycin resistance cassette (Puro^R^), and the microRNA scaffold ultramer containing the individual sense and antisense sequence. Primary primers (black) were used to enriched for the shRNA construct. Secondary primers recognition sequence (red) binds to the exogenous mir30 region of shRNA construct while adding sequence (green) for the barcoded tertiary primers to bind to. Tertiary primer: recognition sequence binds to the previous amplicon (green) while adding barcode and Illumina sequencing tag (blue).

A lentivirus shRNA library containing 113,001 shRNAs covering 18,938 genes with an average of 6 shRNAs per gene was purchased from transOMIC technologies inc. Individual shRNAs were randomly spread across 13 pools. Each pZIP lentiviral vector contained the hCMV promoter driving expression of a ZsGreen cassette, a puromycin resistance cassette, and the microRNA scaffold Ultramer containing the individual shRNA (Figure 1B). Viral packaging was performed by the Viral Vector Core at the University of Iowa and the final concentrated and titered library was sequence-confirmed.

The 13 pools of viruses (obtained from the Viral Vector Core Facility, University of Iowa) were transduced into 3.6 × 10^7^ A375 cells at an MOI of 0.5 TU/cell. For each pool of lentivirus, cells were divided equally into 12 wells and incubated overnight before being pooled back together and split into two flasks. Two days later, one of the two flasks was treated with DMSO and the other with Vemurafenib at 3 μM. A few days later, each flask was divided into three flasks and treated with DMSO or Vemurafenib 3 μM for three and 21 days, respectively, at which time point the flasks reached confluency. Genomic DNA was extracted via GenElute^TM^ Mammalian Genome DNA miniprep Kit (Sigma). DNA fragments containing the shRNA constructs were amplified three times (Table 1). The first PCR was performed with primers recognizing the puromycin resistance cassette and the WPRE (woodchuck hepatitis virus post-transcriptional regulatory element), both found exclusively in the insert lentivirus construct. This step is necessary to enrich for the shRNA construct since the secondary PCR primers also partially bind to endogenous mir30. The secondary PCR amplifies the shRNA sequence and the tertiary PCR barcodes each sample and tags each amplicon for sequencing via the Illumina HI-Seq 4000 platform.

**Table 1:** List of primers.

**Primary PCR amplicon library preparation**
Primary shRNA (Puro): GCAACCTCCCCTTCTACGAG
Primary shRNA (WPRE): GGCATTAAAGCAGCGTATCC
**Secondary PCR amplicon library preparation**
shRNAfor_V1.1: ACACTGACGACATGGTTCTACAAATCGTTGCCTGCACATCTT
shRNAfor_V1.2: ACACTGACGACATGGTTCTACAKAATCGTTGCCTGCACATCTT
shRNAfor_V1.3: ACACTGACGACATGGTTCTACANKAATCGTTGCCTGCACATCTT
shRNAfor_V1.4: ACACTGACGACATGGTTCTACANKNAATCGTTGCCTGCACATCTT
shRNAfor_V1.5: ACACTGACGACATGGTTCTACANNGRAATCGTTGCCTGCACATCTT
shRNAfor_V1.6: ACACTGACGACATGGTTCTACANNNGRAATCGTTGCCTGCACATCTT
shRNAfor_V2.1: ACACTGACGACATGGTTCTACACCTTGAATTCCGAGGCAGTA
shRNAfor_V2.2: ACACTGACGACATGGTTCTACAKCCTTGAATTCCGAGGCAGTA
shRNAfor_V2.3: ACACTGACGACATGGTTCTACANKCCTTGAATTCCGAGGCAGTA
shRNAfor_V2.4: ACACTGACGACATGGTTCTACANKNCCTTGAATTCCGAGGCAGTA
shRNAfor_V2.5: ACACTGACGACATGGTTCTACANNGRCCTTGAATTCCGAGGCAGTA
shRNAfor_V2.6: ACACTGACGACATGGTTCTACANNNGRCCTTGAATTCCGAGGCAGTA
shRNArev_V1.1: TACGGTAGCAGAGACTTGGTCTAATCGTTGCCTGCACATCTT
shRNArev_V1.2: TACGGTAGCAGAGACTTGGTCTKAATCGTTGCCTGCACATCTT
shRNArev_V1.3: TACGGTAGCAGAGACTTGGTCTNKAATCGTTGCCTGCACATCTT
shRNArev_V1.4: TACGGTAGCAGAGACTTGGTCTNKNAATCGTTGCCTGCACATCTT
shRNArev_V1.5: TACGGTAGCAGAGACTTGGTCTNNGRAATCGTTGCCTGCACATCTT
shRNArev_V1.6: TACGGTAGCAGAGACTTGGTCTNNNGRAATCGTTGCCTGCACATCTT
shRNArev_V2.1: TACGGTAGCAGAGACTTGGTCTCCTTGAATTCCGAGGCAGTA
shRNArev_V2.2: TACGGTAGCAGAGACTTGGTCTKCCTTGAATTCCGAGGCAGTA
shRNArev_V2.3: TACGGTAGCAGAGACTTGGTCTNKCCTTGAATTCCGAGGCAGTA
shRNArev_V2.4: TACGGTAGCAGAGACTTGGTCTNKNCCTTGAATTCCGAGGCAGTA
shRNArev_V2.5: TACGGTAGCAGAGACTTGGTCTNNGRCCTTGAATTCCGAGGCAGTA
shRNArev_V2.6: TACGGTAGCAGAGACTTGGTCTNNNGRCCTTGAATTCCGAGGCAGTA
**Tertiary PCR amplicon library preparation**
(FLUDIGM ACCESS ARRAY BARCODED PRIMERS PRODUCT# 100-4876)
**Gene expression quantification**
CUL3for: ACGACAGGATATTGGCCCAC
CUL3rev: ATGCTGGAGTGTGAGCTGTC
ERBB3for: CACAATGCCGACCTCTCCTT
ERBB3rev: ATCGTAGACCTGGGTCCCTC
FADS2for: CCCCTGCTGATTGGTGAACT
FADS2rev: CTCTCCAGGGCGATGATGTG
FJX1for: TCCCACGCTGTTTCCTTTCA
FJX1rev: CCCAAGAATGGGGTGCATCT
GAPDHfor: CCATGTTCGTCATGGGTGTG
GAPDHrev: CAGGGGTGCTAAGCAGTTGG
RAC1for: AAACCGGTGAATCTGGGCTT
RAC1rev: TGATGCAGGACTCACAAGGG
RhGDIfor: CTGCACACCAGGGTCAGG
RhGDIrev: ACGAGAGCCTGCGAAAGTAC
TAOK1for: CAGCCTGAAGGACCCTGAAAT
TAOK1rev: CCACCACTTCATTGGTACGC
TBPfor: TTCGGAGAGTTCTGGGATTG
TBPrev: CTCATGATTACCGCAGCAAA
TFfor: ACGGGAGGTCAAAGATTGCG
TFrev: ATCAGGGACAGCCAGACACA
ZYG11Bfor: ACAAAAAGACATCCTACCTAACCT
ZYG11Brev: TCATTGGCTTCCCCAGACAC

### Screen Analysis

Reads were aligned to target sequences belonging to each shRNA construct used in the screen. Alignment was performed using HISAT2 (14). Only reads that mapped uniquely and had at most one mismatch were considered for further analysis. Final counts reported for a given tag were the raw number of reads normalized by the total number of reads in a given sample, i.e., counts per million. Candidates were selected for validation based on the log2 fold change. Statistical significance was determined using the Mann Whitney U-test. This analysis was implemented using the R programming language.

### Hit Validation

A375 cells were transduced with lentivirus shRNA targeting each of the nine genes selected for validation. Two to four shRNA guides (transOMIC technologies inc.), including those recovered in the screen, were used to target each gene independently (Table 2). 293FT cells were transfected with 1 μg lentiviral shRNA vector, 0.1 μg VSV-G and 0.9 μg PAX2 using Polyfect (Qiagen). Forty-eight hours later, the viral suspension was collected and added to A375 cells along with polybrene (8 μg/ml final). Puromycin (1 μg/ml) was added to the media 2 days after the infection to select for transduced cells. Transduction efficiency was evaluated by FACS (expression of ZsGreen) and knockdown efficiency was evaluated by RT-PCR and/or western blot.

**Table 2:** shRNA construct IDs.

	**shRNA construct ID**
NF1 #1	ULTRA-3327856
NF1 #2	ULTRA-3327858
NF1 #3	RRUH-156067
NF1 #4	ULTRA-3327857
SUV420H1 #1	RRUH-149060
SUV420H1 #2	RRUH-114717
SUV420H1 #3	ULTRA-3335298
SUV420H1 #4	RRUH-150488
TAOK1 #1	ULTRA-3354195
TAOK1 #2	RRUH-153562
TAOK1 #3	RRUH-172375
CUL3 #1	RRUH-103948
CUL3 #2	ULTRA-3405220
ERBB3 #1	ULTRA-3238012
ERBB3 #2	ULTRA-3238013
ERBB3 #3	ULTRA-3238014
ERBB3 #4	RRUH-105182
TF #1	ULTRA-3380716
TF #2	ULTRA-3380718
TF #3	ULTRA-3380720
TF #4	ULTRA-3483029
FJX1 #1	ULTRA-3257832
FJX1 #2	ULTRA-3257833
FJX1 #3	ULTRA-3257835
FJX1 #4	ULTRA-3257836
FADS2 #1	ULTRA-3414820
FADS2 #2	ULTRA-3414821
FADS2 #3	ULTRA-3414823
FADS2 #4	ULTRA-3414819
ZYG11B #1	ULTRA-3395524
ZYG11B #2	ULTRA-3395526
ZYG11B #3	ULTRA-3395527
ZYG11B #4	RRUH-160969

*List of shRNA IDs obtained from transOMIC technologies inc. The constructs recovered in the screen are indicated in red*.

### A375 CUL3 and RAC1 Double KD

A375 shNT, A375 shCUL3#1 and A375 shCUL3#2 cells were transduced with retrovirus shRNA targeting RAC1 (transOMIC technologies inc.). GP2-293 cells were transfected with 1 μg pSIREN Hygro ShRNA vector and 1 μg VSV-G using Polyfect (Qiagen). Forty-eight hours later, the viral suspension was collected and added to A375 cells along with polybrene (8 μg/ml final). Puromycin (1 μg/ml) and hygromycin (400 μg/ml) were added to the media 2 days after the infection to select for transduced cells. Knockdown efficiency was evaluated by western blot.

### RT-PCR

RNA was extracted using RNeasy Mini Kit (Qiagen, cat # 74104). Retrotranscription was performed on 1 μg of RNA using iScript cDNA Synthesis Kit (Bio-Rad, 170-8890). Real-time amplification was performed with the CFX Connect Real-Time System (Biorad) using iQ SYBR Green Supermix (Bio-Rad, 170-8880) (Table 1).

### Viability Assays

For short-term IC_50_ assays, cells were plated at 1,500 cells/well in 96-well plates and treated the following day with 10-fold serial dilutions of vemurafenib (1 nM−10 μM). After 72 h, cell viability was determined using Cell-Titer-Blue assay (Promega) according to manufacturer's instructions. The Synergy HT plate reader (Biotek, Winooski, VT) was used for signal quantification and the fluorescence values were normalized to the vehicle well for each cell line. Absolute IC50 were calculated with GraphPadPrism software (GraphPad Software, Inc.).

For short term growth assays, cells were plated in triplicate in 96-well plates at a density of 5 × 10^2^ cells per well. The CellTiter-Blue Viability Assay (Promega) was performed serially on pre- and post-inhibitor treated cells. Media containing the specific inhibitor used was renewed every 5 days. Fold change from Day 0 was assessed for each well by comparing pre- and post-inhibitor treated cells.

The effect of 4 days treatment with vemurafenib, saracatinib and the combination vem/sara on cell death and cell cycle progression was evaluated by flow cytometry. Cells were harvested, washed with PBS and either directly stained with propidium iodide 1 μg/ml (cell death) or fixed with ethanol 70% overnight at −20°C prior staining with propidium iodide 35 μg/ml (cell cycle analysis).

For long term growth assays, cells were maintained on vemurafenib (3 μM) or DMSO for 70 days. Every 5 days, the cells were passaged, counted and the population doubling level (PDL) was calculated using the formula: PDLn = 3.32 (log Xt–log X0) + PDLn-1 (with Xt = cell number at that point, X0 = cell number used as inoculum and PDLn-1 = population doubling level at the previous passage). Vemurafenib treatment was renewed at each passage.

### Western Blot

Proteins were extracted with Laemmli buffer, separated on SDS-polyacrylamide gels (NuPAGE® 4–12% Bis-Tris Protein Gels, Novex) and transferred to PVDF membranes (Immobilon-FL) prior to incubation with primary antibody (1/1,000) overnight at 4°C and incubation with corresponding secondary antibody (1/10,000) for 1 h at room temperature (Table 3). The membranes were scanned with an Odyssey infrared imaging system (LI-COR Biosciences, Lincoln NE) and expression of protein was quantified (ImageJ) and normalized to β-actin or α-tubulin expression.

**Table 3:** List of antibodies.

**Primary antibodies**
RAC1 (#610651, BD Transduction)
CDC42 (#2462, Cell Signaling)
RHOA (#ARH03, Cytoskeleton)
RHOC (#3430S, Cell Signaling)
RHOGDI (#2564S, Cell Signaling)
p44/42 MAPK (ERK1/2) (#9102, Cell Signaling Technologies)
phospho-p44/42 MAPK (T202/Y204) (#9101, Cell Signaling Technologies)
MEK1 (#2352, Cell Signaling Technologies)
phospho-MEK1 (Ser217) (#9154, Cell Signaling Technologies)
phospho-MEK1 (Ser298) (#98195, Cell Signaling Technologies)
β-actin (6221, BioLegend; A1978, Sigma)
α-tubulin (12G10, DSHB)
CUL3 (#2759, Cell Signaling Technologies)
NF1 (#14623, Cell Signaling Technologies)
Cleaved Caspase 3 (Asp175) (#9661, Cell Signaling Technologies)
**Secondary antibodies**
Goat anti-rabbit IRDye 680 RD (#925-68071, LI-COR)
Donkey anti-mouse IRDye 800 CW (#610-731-124, Rockland)
Goat anti-mouse Alexa Fluor 680 (#A21058, Invitrogen)
Goat anti-rabbit Alexa Fluor 790 (#A11369, Invitrogen)

### RAC1 Activity Assay

Cells were released from 10 cm dish with trypsin, washed twice with PBS and lysed using RAC1 activity buffer (50 mM Tris, 500 mM NaCl, 50 mM MgCl_2_, 1% Triton X-100, 0.2 mM PMSF, proteases inhibitor cocktail). To detect GTP-bound RAC1, 800–1,000 μg of protein was incubated with recombinant protein (Rho binding domain of Rhotekin for RhoA/C or of PAK1 for RAC1) (>30 μg) for 45 min at 4°C (5% of the sample was reserved for use as the input control). Samples were washed three times with 1 mL of RAC1 activity buffer and resuspended in 30 μl of loading buffer containing 10% BME. Samples were separated on SDS-polyacrylamide gels to detect RAC1 protein.

## Results

### Pooled shRNA Transduction Drives Vemurafenib Resistance

To identify genes whose down-regulation confers resistance to MAPKi, we transduced A375 human melanoma cells harboring the BRAF^V600E^ mutation with a lentiviral shRNA library consisting of 113,001 shRNAs covering 18,938 genes with an average of six shRNAs per gene at a MOI of 0.5 (Figure 1A). For each of the 13 pools, the transduced cells were split into six flasks cultured either in the presence of DMSO (three flasks) or 3 μM vemurafenib (three flasks) to ensure biological replicates. Cells reached confluence after 3 days for DMSO-treated flasks and 21 days for vemurafenib-treated flasks. Drug-resistant colonies emerged around 2 weeks after initial vemurafenib treatment. Based on literature and our previous work, spontaneous resistance to 3 μM vemurafenib occurs around 3–4 weeks post-treatment, indicating that shRNA knockdown decreased time to resistance.

### Identification of Enriched shRNA Drivers in Vemurafenib Resistant A375

To identify genes in which knockdown via shRNA conferred vemurafenib resistance, the relative abundance of each shRNA was determined by PCR amplification and next generation sequencing of the lentiviral library. To accomplish this, genomic DNA was extracted from 39 DMSO- and 39 vemurafenib-treated cell populations (three flasks per treatment for each of the 13 pools of lentivirus). DNA fragments containing the puromycin resistance cassette and WPRE regions of the lentivirus backbone were amplified to enrich for the exogenous shRNA construct (Figure 1B, Table 1). Subsequent PCR steps barcoded each sample and prepared each fragment for sequencing on the HiSeq 4000 with Fluidigm sequencing primers (Figure 1B, Table 1).

Enriched shRNAs were defined based on criteria including number of fragments per million reads, representation across three independent vemurafenib treated populations, and fold change in comparison to DMSO-treated populations (Supplementary Figure 1). High-performing shRNAs were represented in two groups: strongly enriched (13 genes) and weakly enriched (440 genes). Strongly enriched tags were defined as >50,000 fragments/million normalized reads in all three vemurafenib-treated samples with log_2_ fold-change above eight over DMSO samples. Weakly enriched shRNAs also met the log_2_ fold-change of eight threshold but failed the reads/sample cutoff. This data set was highly complex with many tags failing to have similar enrichment patterns across biological replicates of vemurafenib treated populations. To further filter the genes of interest, expression in A375 was analyzed for each gene, and only genes that had baseline expression in A375 were selected for further study (CCLE, Broad Institute). The final gene list we considered for further validation studies included *TAOK1, SUV420H1, ERBB3, FADS2, FJX1, TF, ZYG11B, NF1*, and *CUL3* (Table 4). Of note, two out of four NF1 shRNA constructs were strongly enriched in our screen. This gene represents a positive control in this study since it has previously been identified as a driver of MAPKi resistance in A375 cells using an shRNA screen (8).

**Table 4:** Filtered list of genes with enrichment in vemurafenib resistant cells.

**Tag**	**Gene Symbol**	**Log2(fold change)**	**Vem 1 (reads/million)**	**Vem 2 (reads/million)**	**Vem 3 (reads/million)**
RRUH-103948	CUL3	11.8	254,065	226,472	201,012
ULTRA-3327857	NF1	8.9	963,636	940,936	953,047
RRUH-156067	NF1	12.2	189,462	119,632	181,222
ULTRA-3483029	TF	10.1	346,248	339,613	146,516
ULTRA-3257836	FJX1	9.7	122,124	92,277	124,709
ULTRA-3414819	FADS2	8.4	136,014	130,292	126,370
RRUH-160969	ZYG11B	8.5	82,434	712,989	498,289
RRUH-105182	ERBB3	11.8	72,086	85,512	81,783
RRUH-149060	SUV420H1	11.8	5,424	29,980	25,460
RRUH-150488	SUV420H1	5.7	22,867	23,233	15,405
RRUH-153562	TAOK1	11.6	21,136	9,241	3,405

*Black, Strongly enriched; Green, Weakly enriched*.

### Loss of NF1 and CUL3 Confers Vemurafenib Resistance

**Figure 2:**
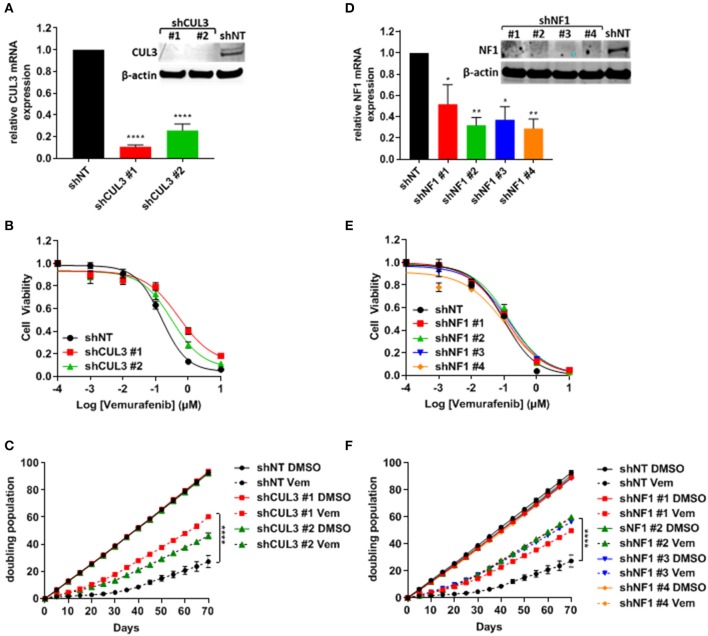
Knockdown of NF1 and CUL3 drive vemurafenib resistance in A375 cells. **(A,D)** The efficiency of CUL3 **(A)** and NF1 (**D)** knockdown in A375 cells was confirmed by RT-PCR and western blot [*p < 0.05; **p < 0.01; ****p < 0.0001 (One-way ANOVA followed by Bonferroni's multiple comparisons test)]. **(B,E)** Sensitivity to vemurafenib was evaluated in short-term (72 h) dose response assay in CUL3 **(B)** and NF1 **(E)** A375 knockdown cells. **(C,F)** Sensitivity to vemurafenib was evaluated in long-term (70 days) growth assay in CUL3 **(C)** and NF1 **(F)** A375 knockdown cells [****p < 0.0001 (Two-way ANOVA followed by Bonferroni's multiple comparisons test)]. *n* ≥ 3 for each experiment. See Supplementary Figure 6 for original blots.

To verify resistance effects of the nine selected candidates, we independently expressed shRNA guides to these nine genes in A375 cells. Each gene was represented by two to four unique shRNAs including the shRNA sequences that were identified by the screen. After puromycin selection, knockdown was confirmed by qRT-PCR and/or immunoblot analysis. Knockdown cell lines were analyzed for short-term response to vemurafenib and growth in long-term culture while undergoing vemurafenib treatment (Figure 2 and Supplementary Figure 2). Despite an effective reduction of mRNA levels (Supplementary Figure 2, left panel), knockdown of TAOK1, SUV420H1, ERBB3, FADS2, FJX1, TF, and ZYG11B all failed to confer resistance to vemurafenib in both short-term (Supplementary Figure 2, middle panel) and long-term assays (Supplementary Figure 2, right panel). Although one ZYG11B shRNA slightly increased the IC_50_ and growth in long-term culture, the three others failed to confer resistance, suggesting that the resistance could be due to an off-target effect. On the other hand, while only CUL3 knockdown increased IC_50_ to vemurafenib in short term culture (159 nM in shNT vs. 589 and 312 nM in shCUL3 #1 and #2, respectively) (Figures 2B,E), all shRNA constructs targeting either CUL3 or NF1 drove resistance to vemurafenib in long-term culture (Figures 2C,F). We also performed the knockdown of CUL3 in a second melanoma cell line, 451.Lu (Supplementary Figure 3A). As expected, knockdown of CUL3 via two independent shRNA constructs drove resistance to vemurafenib in 451.Lu cells shown by an increase of the IC_50_ in a short-term experiment (122 nM in shNT vs. 3223 and 930 nM in shCUL3 #1 and #2, respectively) (Supplementary Figure 3B).

### Vemurafenib Resistance Is Associated With the Reestablishment of MAPK Signaling in CUL3^KD^ Cells

**Figure 3:**
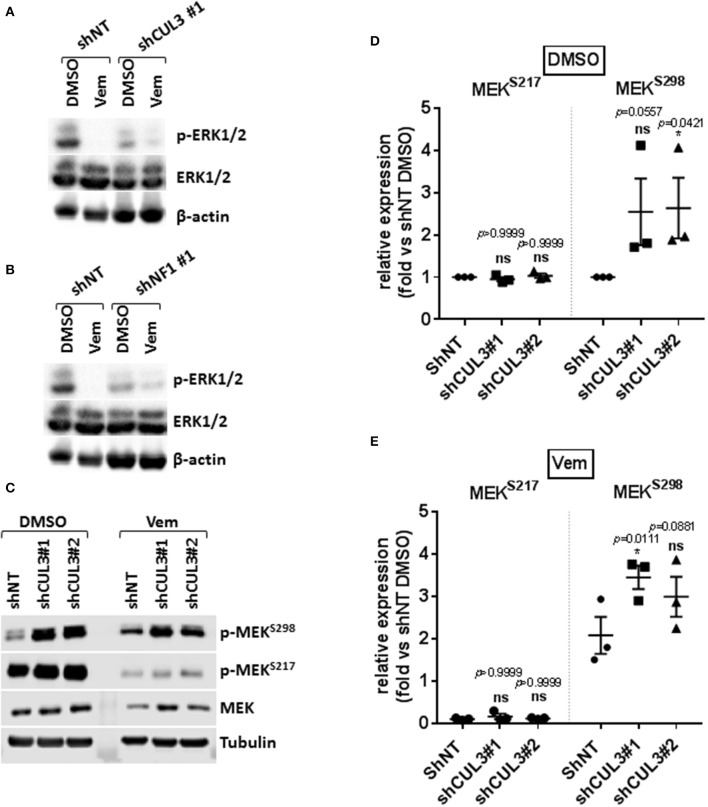
Resistance to vemurafenib is associated with the reestablishment of MAPK signaling in CUL3^KD^ cells. **(A,B)** Western blot of p-ERK1/2 and total ERK1/2 in A375 NF1^KD^ **(A)** and CUL3^KD^ **(B)** cells treated with DMSO or vemurafenib (3 μM) for 18 h. **(C–E)**, Expression **(C)** and quantification **(D,E)** of phospho-MEK^S217^ and phospho-MEK^S298^ measured by western blot in A375 CUL3^KD^ cells treated with DMSO or vemurafenib (3 μM) for 4 days. *p < 0.05 (Two-way ANOVA followed by Bonferroni's multiple comparisons test), *n* = 3. See Supplementary Figures 7, 8 for original blots.

Numerous pathways implicated in driving resistance to vemurafenib have been identified with most mechanisms reestablishing MAPK via phospho-ERK or activating downstream targets of ERK. We demonstrated that resistance to vemurafenib was associated with a sustained activation of the MAPK pathway as shown by the elevated pERK1/2 level in A375 CUL3^KD^ (Figure 3A) and NF1^KD^ (Figure 3B) cells compared to A375 shNT cells after an 18-h treatment with vemurafenib. While the role of NF1 in MAPKi resistance has previously been characterized (8, 15), the mechanism by which loss of CUL3 function contributes to BRAFi-resistance remained unclear despite being previously identified in a CRISPR screen for BRAFi-resistance (12). Therefore, we focused our efforts on determining the underlying mechanisms leading to the reestablishment of MAPK signaling in CUL3^KD^ cells upon vemurafenib treatment. While loss of CUL3 did not affect the phosphorylation of MEK on S217 (RAF-dependent phosphorylation site), it significantly increased the phosphorylation of MEK on S298, a site phosphorylated by PAK1 kinase, in both DMSO- (Figures 3C,D) and vemurafenib-treated conditions (Figures 3C,E). Since PAK1 kinase is a downstream effector of the small GTPase, RAC1, these results suggest that vemurafenib resistance of CUL3^KD^ cells treated with vemurafenib may depend on RAC1 signaling pathways.

### CUL3^KD^ Cells Are Sensitive to Vemurafenib + Srci Treatment

**Figure 4:**
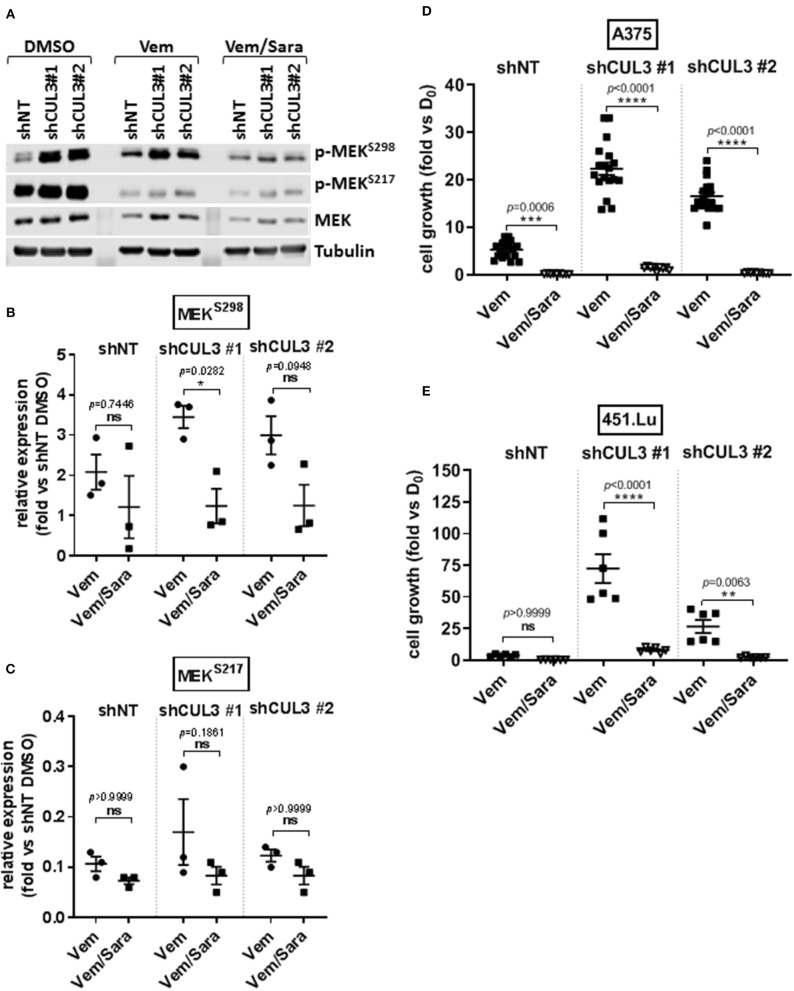
CUL3 Knockdown cells are resistant to Vemurafenib but sensitive to the combination vemurafenib/saracatinib. **(A–C)** Expression **(A)** and quantification of phospho-MEK^S298^ **(B)** and phospho-MEK^S217^ **(C)** measured by western blot in A375 CUL3^KD^ cells treated with vemurafenib (3 μM) alone or in combination with saracatinib (2 μM) for 4 days. **(D,E)** Effect of vemurafenib (3 μM) alone or in combination with saracatinib (2 μM) on the growth of A375 **(D)** or 451.Lu **(E)** CUL3^KD^ cells (10 days treatment). *p < 0.05; **p < 0.01; ***p < 0.001; ****p < 0.0001 (Two-way ANOVA followed by Bonferroni's multiple comparisons test), *n* ≥ 3 for each experiment. See Supplementary Figure 8 for original blots.

**Figure 5:**
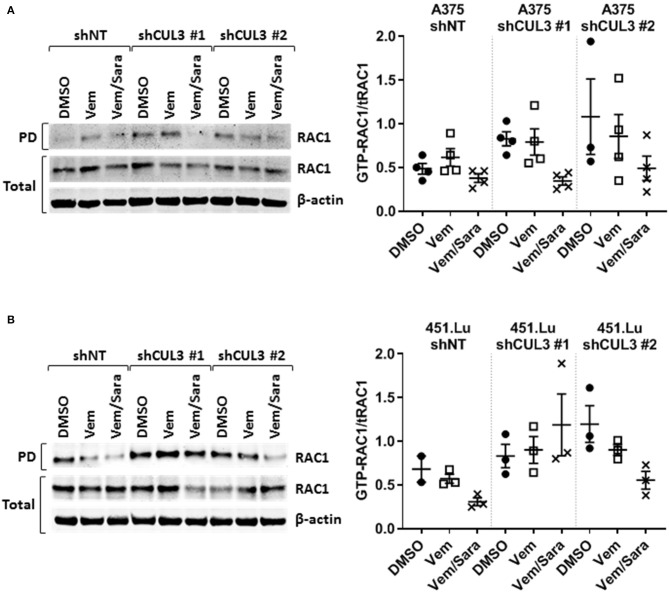
RAC1 activity is increased in CUL3^KD^ cells and inhibited by the combination vemurafenib/saracatinib. Activity of RAC1 was assessed by pulldown of GTP-RAC1 followed by immunoblotting of RAC1 in **(A)** A375 and **(B)** 451.Lu CUL3^KD^ cells. See Supplementary Figures 9, 10 for original blots.

Activated Rho-GTPases have been shown to play an important role in MAPKi resistance. Indeed, constitutively active mutant of *RAC1* (*RAC1*^*P*29*S*^) is found in 10% of MAPKi progression melanoma tumors and overexpression of PAK may promote MAPKi resistance in some settings (9, 10, 16–18). Based on our previous work showing that RAC-driven mechanisms of resistance can be ablated with the addition of the Src family inhibitor (Srci) saracatinib (19), we evaluated the effect of vemurafenib in combination with saracatinib. The drug combination decreased the level of pMEK^S298^ in both A375^NT^ and CUL3^KD^ cells compared to vemurafenib alone (Figures 4A,B) whereas it had no effect on pMEK^S217^ level (Figures 4A,C). While saracatinib had minimal (A375) or no (451.Lu) effect on its own (Supplementary Figures 4A,B), it had a cytotoxic effect when combined with vemurafenib on A375 CUL3^KD^ (Figure 4D) and 451.Lu CUL3^KD^ (Figure 4E) cells. To confirm the cytotoxic effect of combined vemurafenib/saracatinib treatment, we evaluated the percentage of cell death by staining treated cells with propidium iodide (Supplementary Figure 4C). As anticipated, saracatinib alone did not induce cell death while vemurafenib induced cell death in A375 shNT and to a lesser extent in A375 shCUL3 #1 and #2. However, the vemurafenib/saracatinib combination dramatically increased the percentage of dead cells in all cell lines evaluated. These results were consistent with induction of caspase three cleavage upon vemurafenib or combined vemurafenib/saracatinib (Supplementary Figures 4D,E). Additionally, we investigated the effect of the different treatment on cell cycle progression (Supplementary Figure 4F). Our results indicated that saracatinib on its own does not affect cell cycle progression while vemurafenib induced a cell cycle arrest with an accumulation of cells in G1. However, the addition of saracatinib to vemurafenib does not exacerbate this cell cycle arrest suggesting that the drug combination acts through cell death induction. Finally, RAC1 pull-down activation assays showed increased basal RAC1 activity in A375 (Figure 5A) and 451.Lu (Figure 5B) CUL3^KD^ cells compared to their respective control cells. While the activity of RAC1 remained unchanged upon vemurafenib treatment, it was decreased when cells were treated with vemurafenib plus saracatinib (Figures 5A,B). Thus, these results indicate that the resistance mechanisms driven by knockdown of CUL3 involve the reestablishment of MAPK signaling as a result of the stabilization and Src-dependent activation of RAC1.

### CUL3^KD^ Does Not Affect RAC1 Expression

**Figure 6:**
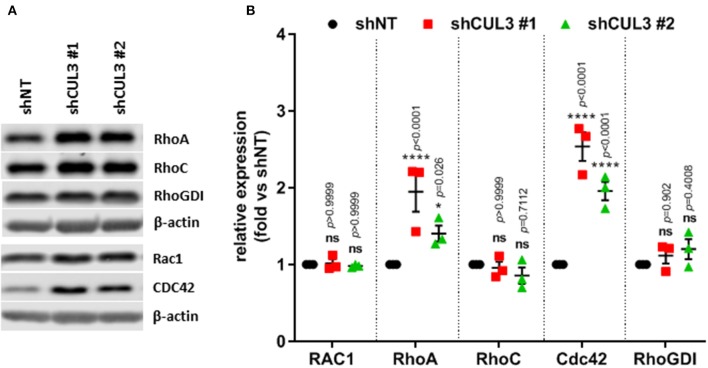
CUL3^KD^ increases expression of RhoA and Cdc42, while having no effect on RhoC, RAC1, and RhoGDI. **(A)** Expression and quantification **(B)** of RhoGTPases (RhoA, RhoC, RAC1, and CDC42) and RhoGDI measured by western blot in A375 CUL3^KD^ cells. *p < 0.05; ****p < 0.0001 (Two-way ANOVA followed by Bonferroni's multiple comparisons test), *n* = 3. See Supplementary Figure 11 for original blots.

CUL3 is a component of multiple E3 ubiquitin-protein ligase complexes. Cullin proteins serve as a scaffold to connect two functional modules of E3 ubiquitin ligases: the catalytic RING-box protein 1 (RBX1), and a Bric-a-brac/Tramtrack/Broad (BTB) protein which act as an adaptor and substrate recognition subunit and confer specificity to the E3 ubiquitin ligase (20). Thus, loss of CUL3 would be predicted to stabilize many proteins by preventing E3 ubiquitin ligase-mediated degradation. Among others, it was demonstrated that a CUL3 E3 ligase complex, involving the adaptor BACURD, could mediate degradation of Rho GTPases which are known mediators of MAPKi resistance (21). However, while CUL3^KD^ was associated with increased expression of RHOA and CDC42, the levels of RHOC and RAC1 remained unchanged (Figures 6A,B). Alternatively, CUL3 KD could affect RAC1 activity indirectly. For example, CUL3 could mediate the degradation of RHOGDI, and consequently destabilize RhoGTPases, as RHOGDI was shown to interact with CUL3-based E3 ligases (22). However, the expression of RhoGDI was unaffected by CUL3^KD^ (Figures 6A,B). These results indicate that the loss of CUL3 does not promote the activation of RAC1 through its direct or indirect stabilization, suggesting that CUL3 is involved in the stabilization of upstream regulators of RAC1.

### RAC1^KD^ Partially Reverses Vemurafenib Resistance in A375 CUL3^KD^ Cells

**Figure 7:**
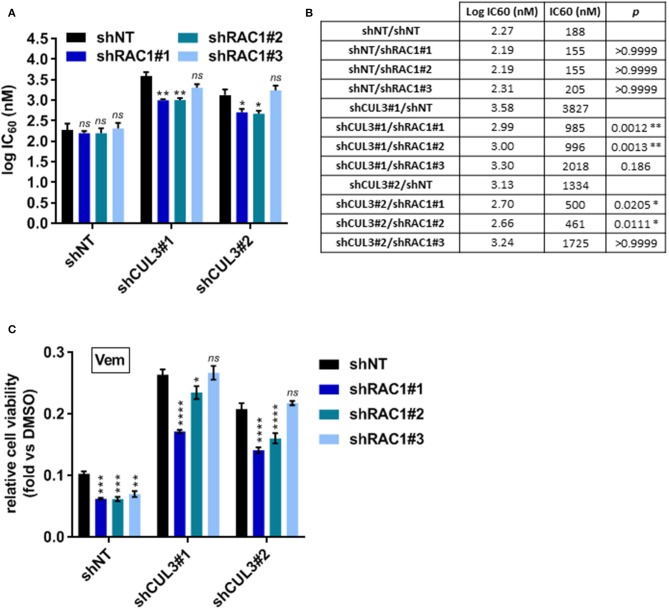
RAC1^KD^ partially reverses vemurafenib resistance in A375 CUL3^KD^ cells. **(A,B)** Sensitivity of RAC1 CUL3 double KD cells to vemurafenib was evaluated in short-term (72 h) dose response assay (1 nM−10 μM). Log IC_60_ were calculated and plotted for each cell lines **(A)**. Log IC_60_ and IC_60_ values are summarized in **(B)**. **(C)** Effect of vemurafenib (3 μM) alone or in combination with saracatinib (2 μM) on the growth of A375 cell line derivatives (5 days treatment). *p < 0.05; **p < 0.01; ***p < 0.001; ****p < 0.0001 (Two-way ANOVA followed by Bonferroni's multiple comparisons test), *n*=3.

To evaluate the implication of RAC1 in the vemurafenib resistance mechanism in CUL3 KD cells, we developed A375 double knockdown for both RAC1 and CUL3. While their efficiency remained modest, the constructs shRAC1#1 and shRAC1#2 appeared to perform better at knocking down RAC1 than shRAC1#3 in A375 CUL3^KD^ cells (Supplementary Figures 5A,B). The knockdown status of CUL3 in these derivatives was also confirmed by western blot (Supplementary Figures 5A,C). The partial loss of RAC1 in the A375 shNT cells did not affect their sensitivity to vemurafenib in a 72h dose response assay (Figures 7A,B and Supplementary Figure 5D). However, RAC1 knockdown increased sensitivity of these cells to vemurafenib (3 μM) exposure in a 5 day growth assay, consistent with previously reported results (19) (Figure 7C). In contrast, expression of the shRAC1#1 and shRAC1#2 constructs resulted in a moderate increase in vemurafenib sensitivity in A375 CUL3^KD^ cells with both assays (Figures 7A–C and Supplementary Figures 5E,F). As noted above, shRAC1#3 was the least potent construct at knocking down RAC1 in A375 CUL3^KD^ cells assay consistent with a a lack of effect in both short- and long-term assays. These results indicate that RAC1 is involved in CUL3-dependendent vemurafenib resistance.

## Discussion

Here we used an shRNA library to identify negative regulators of resistance to vemurafenib in the BRAF^V600E^ -expressing human melanoma cell line A375. While the screen identified multiple genes as candidate drivers, seven out of nine genes tested did not recapitulate the MAPKi resistant phenotype (Table 4 and Supplementary Figure 2). Thus, despite applying stringent criteria for high confidence hits in the screen, the false positive rate was high. This could reflect combinatorial interactions among the hits identified or stochastic fluctuations in library elements through the multiple steps involved in library preparation, representation in cells, or sequencing. The two drivers that did validate, NF1 and CUL3 (Figure 2), had already been identified in previous forward genetic screens using CRISPR approaches in A375 cells (12, 13). Of note, a shRNA-based screen only identified NF1 (8). None of our other high confidence hits were identified in either of these screens with the exception of TAOK1 which was identified in one of the CRISPR screens (12). However, the authors performed two screens from independent transductions and identified the loss of TAOK1 as a driver of vemurafenib resistance in only one of them. The lack of reproducibility amongst screens and/or the absence of discovering known drivers of resistance in patient samples can be explained by different factors. First, although all pooled library screens follow a similar screening protocol (23), the multi-step nature of this process directly affects the quality of the data. Also, additional steps can be added such as antibiotic selection after target cell infection to enrich for infected cell number, though this may constitute an artificial survival selection. Secondly, these screens have been performed in a single melanoma cell line. Since melanoma is a highly heterogeneous disease, the use of a single cell line may limit the possibility of identifying novel resistance mechanisms. Finally, these screens have employed relatively low doses of vemurafenib. While it is difficult to model the pharmacodynamics of vemurafenib exposure in cell culture that melanoma cells experience in patients, prior work has demonstrated that both plasma and intratumoral doses are significantly higher than those conventionally used in cell culture (24). Additionally, low doses of vemurafenib only have a cytostatic effect on A375 cells, while ninety percent of patients experience tumor regression on BRAFi therapy. Therefore, understanding shared or novel resistance mechanisms across cell lines and patient samples at pharmacologically relevant doses is crucial in understanding how to prevent BRAFi resistance.

Most mechanisms implicated in driving BRAFi resistance have been associated with the reactivation of MAPK signaling. NF1, which encodes neurofibromin, is a tumor suppressor which negatively regulates RAS proteins by converting active GTP-bound RAS into the inactive GDP-bound state. Since RAS is an upstream regulator of RAF proteins, how NF1 loss confers resistance to vemurafenib through sustained MAPK pathway activation is well understood (8). How the loss of CUL3 contributes to BRAFi resistance in melanoma is less clear. CUL3 mutation or down-regulation is observed in different types of cancer including lung cancer, oral squamous cell carcinoma and other squamous cell cancers, sporadic PRCC2 (type 2 papillary renal cell carcinoma) and liver cancer (25–28). In addition, a recent study using a transposon mutagenesis screen in mice indicates that CUL3 is a tumor suppressor in lung cancer (29). We show here that the loss of both CUL3 and NF1 were associated with reactivation of the MAPK pathway (Figure 3). More precisely, the loss of CUL3 resulted in increased phosphorylation of MEK on S298, a PAK1-specific phosphorylation site, suggesting that loss of CUL3 increases the activation of PAK1 (Figures 3C,E). Overexpression of PAK is a known driver of MAPKi resistance, and constitutive activating mutations of RAC1 (RAC1^P29S^), an upstream effector of PAK1, are found in 10% of MAPKi progression melanoma tumors (9, 10, 16–18). Our previous work showed that RAC-driven mechanisms of vemurafenib resistance can be ablated with the addition of the Src inhibitor saracatinib (19). We evaluated the effect of this combination of vemurafenib and saracatinib on CUL3^KD^ cells (19). The addition of saracatinib decreased pMEK^S298^ level and abolished vemurafenib resistance in CUL3^KD^ cells (Figure 4). While the exact mechanism of action is still unclear, the efficacy of the drug combination relies on its ability to induce cell death rather than inducing a cell cycle arrest (Supplementary Figure 4). As we previously showed, the efficacy of Srci relied on the ability of Src family proteins to activate GEFs to drive MAPKi resistance (19, 30, 31). This is consistent with the fact that the combination vemurafenib plus saracatinib was able to decrease RAC1 activity, which was elevated in basal and vemurafenib-treated condition in CUL3^KD^ vs. CUL3^NT^ cells (Figure 5). Prior studies have implicated Src in mediating vemurafenib resistance (10, 32–34). However, Src may function both upstream and downstream of RAC1 in some settings, where the two signaling proteins mutually regulate each other (35). Therefore, the ability of Src family inhibition to prevent various resistance mechanisms is most likely due to the ability of Src to participate in many pathways driving resistance. However, because saracatinib is a pan-Src inhibitor, we do not know which Src proteins are important for mitigating MAPKi resistance. As Src is rarely the primary driver of tumorigenesis, Src inhibitors, including saracatinib, have shown little activity in monotherapy trials in solid tumor malignancies (36). Saracatinib as a single agent to treat metastatic melanoma demonstrated no effect, but its activity against MAPKi-resistant melanoma has not been tested (37). Our results presented here support our previous findings identifying the combination BRAFi plus Srci as a viable mechanism to treat RAC1-driven MAPKi resistance mechanisms (19).

Our results do not yet directly reveal the CUL3 substrate that mediates BRAFi resistance. CUL3 plays a critical role in the polyubiquitination and subsequent degradation of specific protein substrates as part of an E3 ubiquitin ligase complex. CUL3 Speckle type BTB/POZ protein (SPOP), kelch-like ECH-associated protein (Keap1) and kelch-like family member 20 (KLHL2.0), being the most representative cancer-associated adaptors of CUL3 (38). The transcription factor NRF2, which promotes cell survival following oxidative damage, is one of the best described targets of CUL3 in the context of cancer through the interaction of CUL3 with the BTB/POZ domain protein Keap1 (39–41). An increase of Nrf2 pathway activation, resulting from the disruption of KEAP1/CUL3/RBX1 E3-ubiquitin ligase complex is observed in many cancers and potentially leads to chemoresistance (28, 42–47). Thus, Nrf2 is an attractive candidate for further study in the context of BRAFi resistance. Alteration of ubiquitinating/deubiquitinating enzymes have been implicated in BRAFi resistance. For example, downregulation of the ubiquitin ligase RNF125 was shown to be involved in intrinsic and adaptive resistance to BRAFi in melanomas through the inhibition of JAK/STAT signaling (48). However, we did not observe alteration of JAK/STAT signaling in our CUL3 KD cells (data not shown). Interestingly, the same authors also identified loss of RBX1 as a driver of vemurafenib resistance. Finally, the loss of USP28, a deubiquitinating enzyme, was shown to stabilize BRAF thus promoting resistance to RAF inhibitor therapy (49).

Our results indicate that CUL3 regulates the activity of RAC1 (Figure 5). Rho GTPases have been shown to be targeted by the CUL3–BACURD E3 ubiquitin ligase for degradation (21). While the role of RAC1 in BRAFi resistance is evident, the role of RHOA, RHOC, and their downstream effector Rho-kinase (ROCK) is less clear with conflicting evidence as to whether ROCK inhibition promotes or inhibits MAPKi in melanoma, and our recent study showed that, in contrast to RAC1 knockdown, which sensitized A375 cells to vemurafenib treatment, knockdown of either RHOA or CDC42 had no such effect (16, 18, 19, 50). Our results indicated that the loss of CUL3 did not affect the expression of RAC1, suggesting that CUL3 does not target RAC1 directly (Figure 6). RhoGDI, which controls the homeostasis of Rho proteins, has been shown to interact with CUL3-based E3 ligases (22, 51). However, our results indicate that CUL3 does not modulate RAC1 activity by targeting RhoGDI, as CUL3^KD^ did not change the expression of RhoGDI (Figure 6). While our results indicate that the loss of CUL3 leads to the activation of RAC1 in melanoma cells and confers resistance to BRAFi, the exact mechanism leading to RAC1 activation in CUL3^KD^ cells is still unclear. One possible mechanism is that CUL3 could promote the degradation of GEFs required for the activation of RAC1 as, for example, CUL3 KBTBD6/KBTBD7 ubiquitin ligase was shown to target TIAM1, a RAC1-specific GEF (52). Another possible mechanism might be a ubiquitin ligase complex specifically targeting the GTP-bound form of RAC1. Torrino et al. previously demonstrated that the ubiquitin ligase HACE1 could preferentially lead to the ubiquitination of GTP-bound RAC1 and that its loss resulted in increased GTP-bound RAC1 cellular levels (53).

Using a forward genetic screen, we identified loss of NF1 and CUL3 as drivers of vemurafenib resistance in melanoma, independently confirming results from similar genetic screens. We show here that CUL3 acts to limit RAC1 activation. When the expression of CUL3 is compromised, the activity of RAC1 is enhanced, thus promoting RAC1-dependent BRAF-independent cell growth. These data confirm our prior findings indicating that Src family inhibitors could represent new treatment options to manage RAC1-driven resistance mechanisms to BRAF inhibitors in melanoma.

## Data Availability Statement

All relevant data generated or analyzed for this study are included in the article/Supplementary Material.

## Author Contributions

MV, CF, MM, MH, CS, AD, and RP conceived and designed the study. MV, CF, AV, EZ, TF, LZ, and KH conducted the experiments. MV, CF, EZ, and AD contributed to data analysis. MV and CF wrote the manuscript. MH, CS, AD, and RP supervised the research. All authors read and approved the final manuscript.

### Conflict of Interest

The authors declare that the research was conducted in the absence of any commercial or financial relationships that could be construed as a potential conflict of interest.

## Abbreviations

BACURD: BTB/POZ domain-containing adapter for CUL3-mediated RhoA degradation protein
BME: β-mercaptoethanol
BTB: Bric-a-brac/Tramtrack/Broad
BTK: Bruton's tyrosine kinase
CCLE: Cancer cell line encyclopedia
CDC42: Cell division control protein 42 homolog
CDKN2A: Cyclin-dependent kinase Inhibitor 2A
CRISPR: Clustered regularly interspaced short palindromic repeats
CUL3: Cullin 3
DMSO: Dimethyl sulfoxide
DNA: Deoxyribonucleic acid
DUSP4: Dual specificity protein phosphatase 4
ERBB3: Erb-B2 receptor tyrosine kinase 3
ERK: Extracellular signal-regulated kinase
FACS: Fluorescence-activated cell sorting
FADS2: Fatty acid desaturase 2
FBS: Fetal bovine serum
FJX1: Four-jointed box kinase 1
GDP: Guanosine 5′-diphosphate
GEFs: Guanine nucleotide exchange factors
GTP: Guanosine-5′-triphosphate
HCK: Tyrosine-protein kinase HCK
hCMV: Human cytomegalovirus
HNSCC: Head and neck squamous cell carcinoma
IC50: Half maximal inhibitory concentration
KBTBD6: Kelch repeat and BTB domain containing 6
KBTBD7: Kelch repeat and BTB domain containing 7
Keap1: Kelch-like ECH-associated protein
KLHL20: Kelch-like family member 20
LCK: Lymphocyte-specific protein tyrosine kinase
MAP2K1: Mitogen-activated protein kinase kinase 1 (a.k.a. MEK1)
MAPK: Mitogen-activated protein kinase
MAPKi: MAP kinase pathway inhibitors
MEK: Mitogen-activated protein kinase kinase
MEKi: MEK inhibitor
mRNA: Messenger RNA
MOI: Multiplicity of infection
NEAA: Non-essential amino acid
NF1: Neurofibromin 1
NF2: Neurofibromin 2
NGS: Next-generation sequencing
NRF2: Nuclear factor erythroid 2-related factor 2
ORF: Open reading frame
PAK1: Serine/threonine-protein kinase PAK 1
PAX2: Paired box gene 2
PBS: Phosphate-buffered saline
PDL: Population doubling level
PIK2R2: Phosphoinositol-3 kinase regulatory subunit 2
PRCC2: Type 2 papillary renal cell carcinoma
PTEN: Phosphatase and TENsin homolog
PVDF: Polyvinylidene difluoride
RBX1: RING-box protein 1
RHOA, Ras homolog gene family: member A
RHOC, Ras homolog gene family: member C
RHOGDI: Rho GDP-dissociation inhibitor
RNA: Ribonucleic acid
RT-PCR: Reverse transcription polymerase chain reaction
SAGA: SPT-ADA-GCN5 acetylase
SDS: Sodium dodecyl sulfate
shRNA: Short hairpin RNA
SPOP: speckle type BTB/POZ protein
STAGA: SPT3-TAF(II)31-GCN5L acetylase
SUV420H1: a.k.a. Lysine methyltransferase 5B (KMT5B)
TAOK1: Thousand and one amino acid protein kinase 1
TF: Transferrin
TIAM1: T-cell lymphoma invasion and metastasis-inducing protein 1
VSV-G: Glycoprotein of vesicular stomatitis virus
WPRE: Woodchuck hepatitis virus post-transcriptional regulatory element
ZYG11B: Zyg-11 family member B.
