# Supplementary material for: Functional Genomic Screening Independently Identifies CUL3 as a Mediator of Vemurafenib Resistance via Src-Rac1 Signaling Axis
Source: Front Oncol. 2020 Apr 3;10:442. doi: 10.3389/fonc.2020.00442 (PMC7169429; doi:10.3389/fonc.2020.00442)
Supplement: Supplementary file 1 [file Table_1.DOCX]

**Supplementary Figures**

**Suppl Fig. 1 Loss-of-function screen results. (A)** Volcano plot of tags recovered. The tags belong to one of four groups as indicated by the colors. Grey tags failed to show strong enrichment defined as greater than or equal to 50000 tags in all three drug selected replicates. Orange tags were strongly enriched but not further tested. Yellow tags were strongly enriched but failed experimental validation. Finally, red tags belonged to NF1 and CUL3, which both passed experimental validation. **(B)** List of the shRNA tag highly enriched in vemurafenib treated condition (>50,000 CPM in all 3 samples). P-values were determined using the Mann-Whitney U test.

**Suppl Fig. 2 Knockdown of SUV420H1, TAOK1, ERBB3, FADS2, FJX1, TF, and ZYG11B all failed to confer resistance to vemurafenib in both short and long-term assays.** Knock down confirmation via RT-PCR *(Left panel)*, 72h dose response assay *(Middle panel)* and long-term (70 days) growth assay *(Right panel)* in **(A)** A375 TF^KD^, **(B)** FJX1^KD^, **(C)** FADS2^KD^, **(D)** ZYG11B ^KD^, **(E)** ERBB3^KD^, **(F)** SUV420H1^KD^ and **(G)** TAOK1^KD^ cells.

**Suppl Fig. 3 Knockdown of CUL3 drives Vemurafenib resistance in 451.Lu cells. (A)** The efficiency of CUL3 knockdown in 451.Lu cells was confirmed by RT-PCR and western blot. *= *p*<0.05 (One-way ANOVA followed by Bonferroni's multiple comparisons test). **(B)** Sensitivity to vemurafenib was evaluated in short-term (72h) dose response assay. n≥2 for each experiment. See Suppl Fig. 12 for original blots.

**Suppl Fig. 4 the combination vemurafenib/saracatinib induces cell death. (A,B)** Effect of DMSO and saracatinib (2µM) on the growth of A375 **(A)** or 451.Lu **(B)** CUL3^KD^ cells (10 days treatment). **(C)** Cells treated with vemurafenib (3µM) alone or in combination with saracatinib (2µM) for 4 days were harvested and stained with propidium iodide to evaluate percentage of cell death by flow cytometry. **(D,E)** Expression **(D)** and quantification of cleaved caspase 3 **(E)** measured by western blot in A375 CUL3^KD^ cells treated with vemurafenib (3µM) alone or in combination with saracatinib (2µM) for 4 days. **(F)** Cells treated with vemurafenib (3µM) alone or in combination with saracatinib (2µM) for 4 days were harvested, fixed with ethanol and stained with propidium iodide to evaluate cell cycle progression by flow cytometry. (a≠b≠c with p<0.05 and *= p<0.05; **= p<0.01; ***= p<0.001; **** = p<0.0001 (Two-way ANOVA followed by Bonferroni's multiple comparisons test)), n≥3 for each experiment. See Suppl Fig. 13 for original blots.

**Suppl Fig. 5 RAC1^KD^ partially reverses vemurafenib resistance in A375 CUL3^KD^ cells. (A-C)** The efficiency of RAC1 knockdown **(A,B)** and CUL3 knockdown **(A,C)** in A375 RAC1 and CUL3 double knockdown cells was evaluated by western blot. **(D-F)** The effect of RAC1 knockdown in A375 shNT (D), A375 shCUL3#1 (E) and A375 shCUL3#2 (F) cells on sensitivity to vemurafenib was evaluated in short-term (72h) dose response assay (1nM to 10µM). (*= p<0.05; **= p<0.01; ***= p<0.001; **** = p<0.0001 (Two-way ANOVA followed by Bonferroni's multiple comparisons test)), n=3 for each experiment. See Suppl Fig. 14 for original blots.

**Suppl Fig. 6 Original blot for Fig.2A & 2D.**

**Suppl Fig. 7 Original blot for Fig.3 A & B.**

**Suppl Fig. 8 Original blots for Fig.3C & 4A.**

**Suppl Fig. 9 Original blot for Fig.5A.**

**Suppl Fig. 10 Original blots for Fig.5B.**

**Suppl Fig. 11 Original blots for Fig.6A.**

**Suppl Fig. 12 Original blot for Suppl Fig.3.**

**Suppl Fig. 13 Original blot for Suppl Fig.4.**

**Suppl Fig. 14 Original blot for Suppl Fig.5.**

**
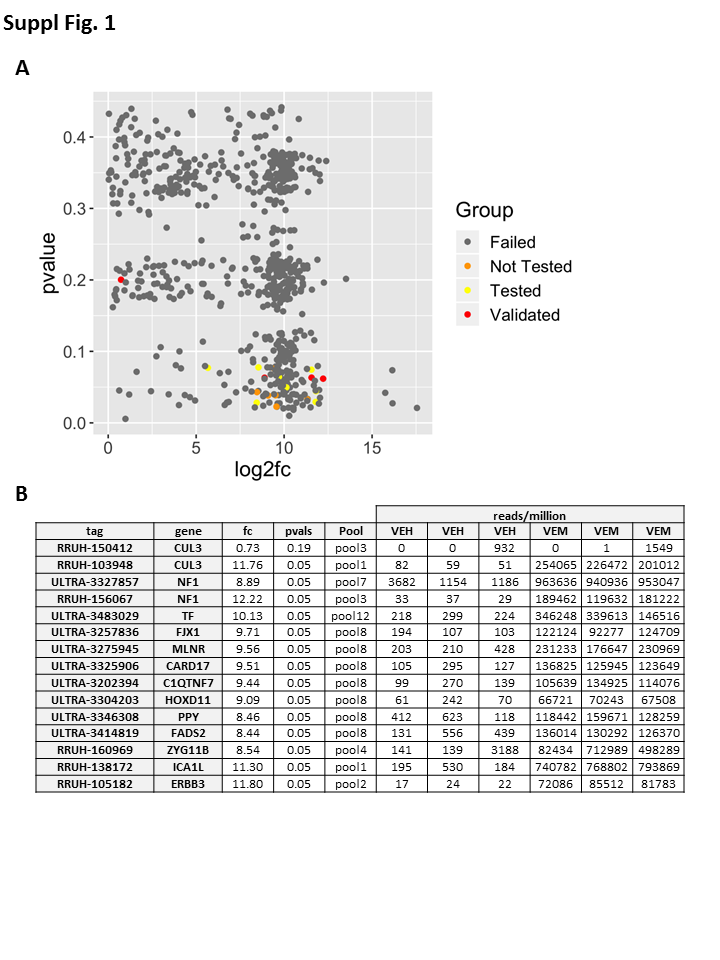
**

**
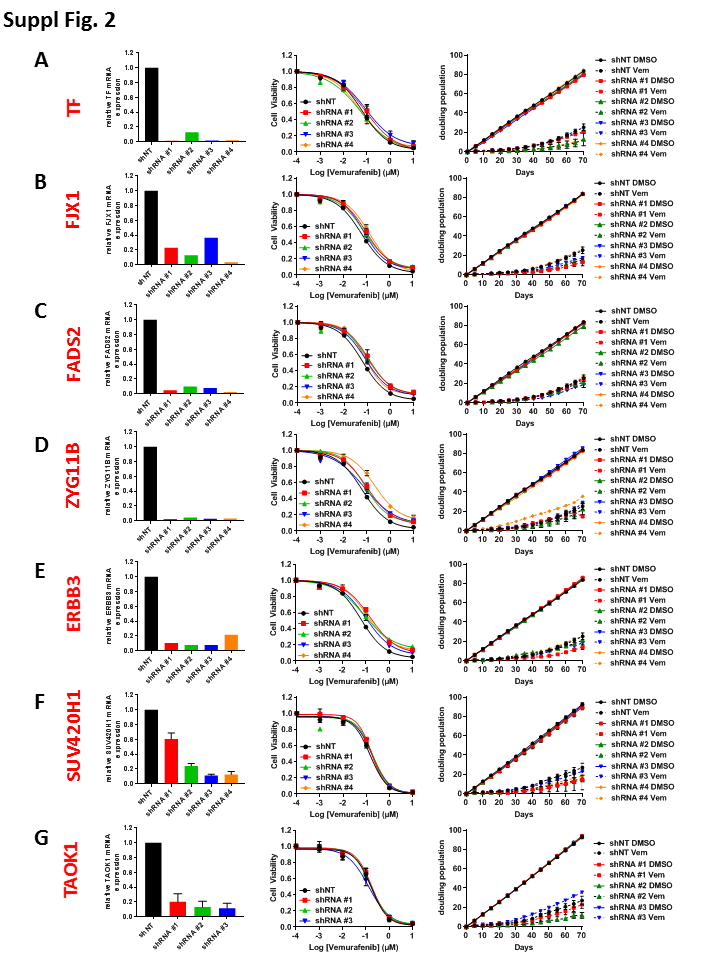

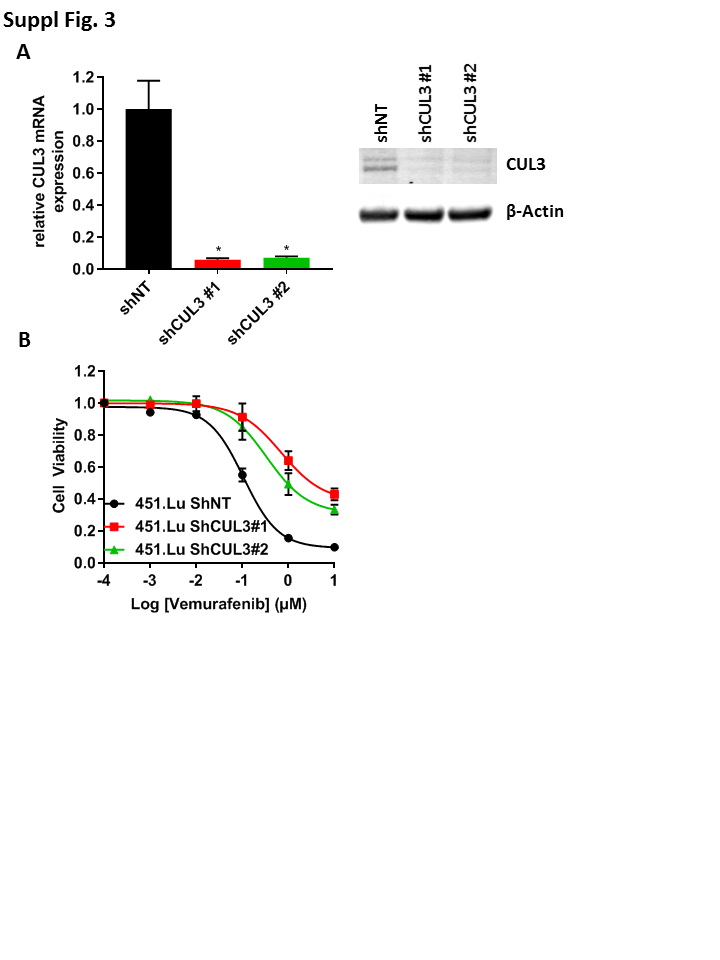

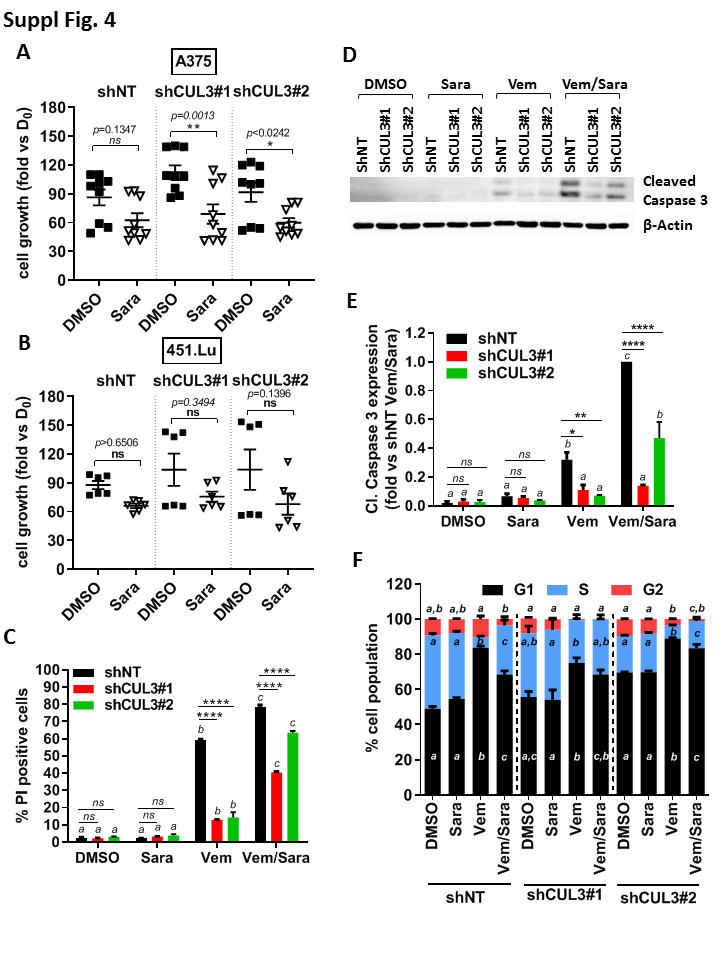
**

**
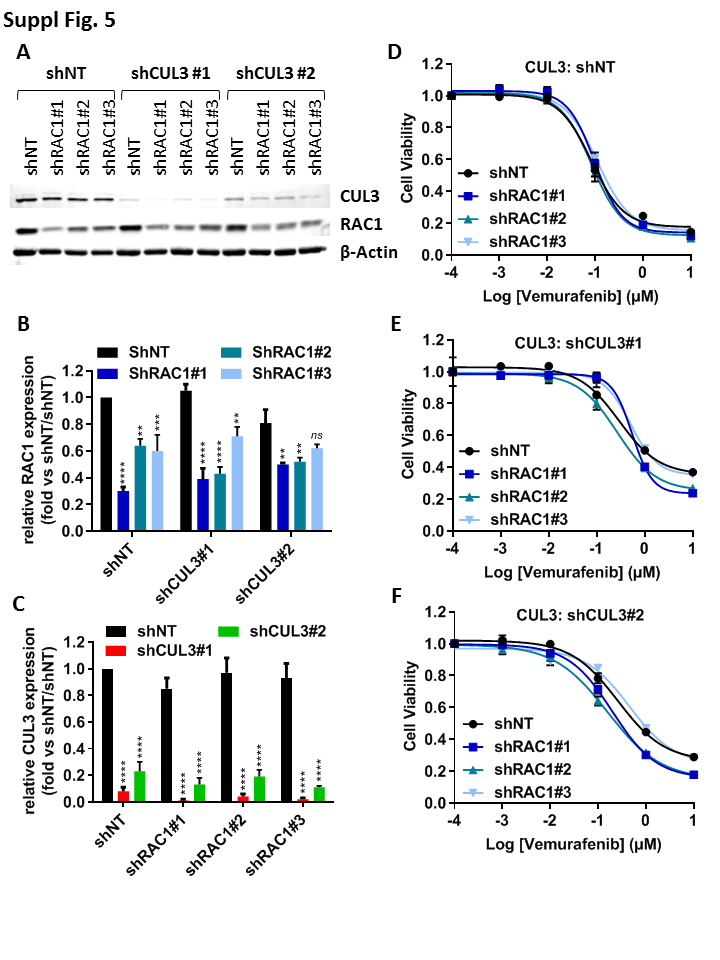
**

**
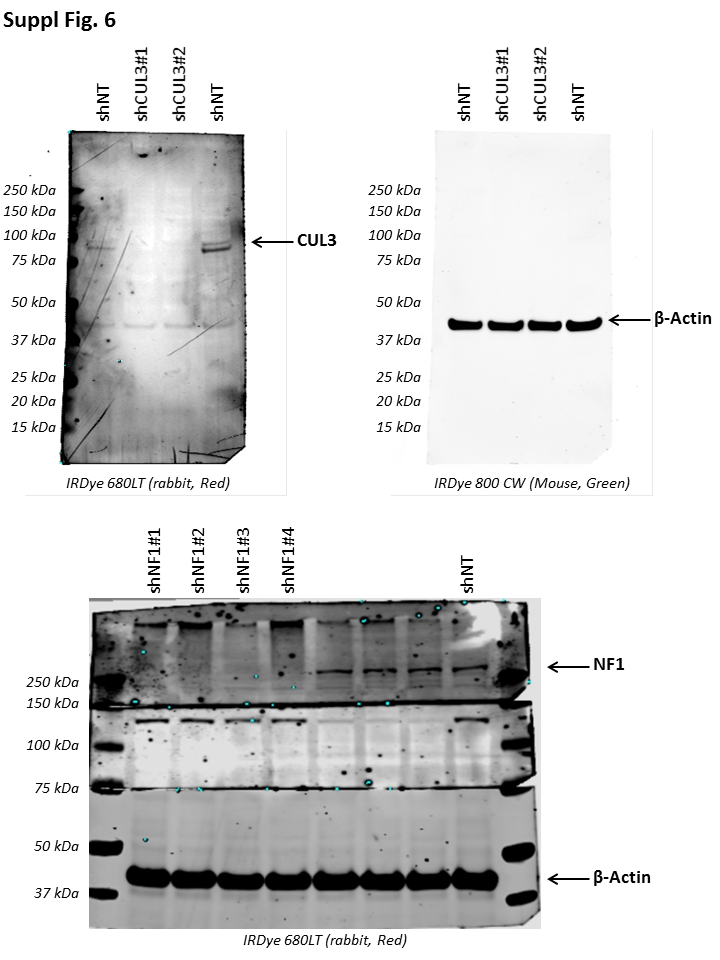
**

**
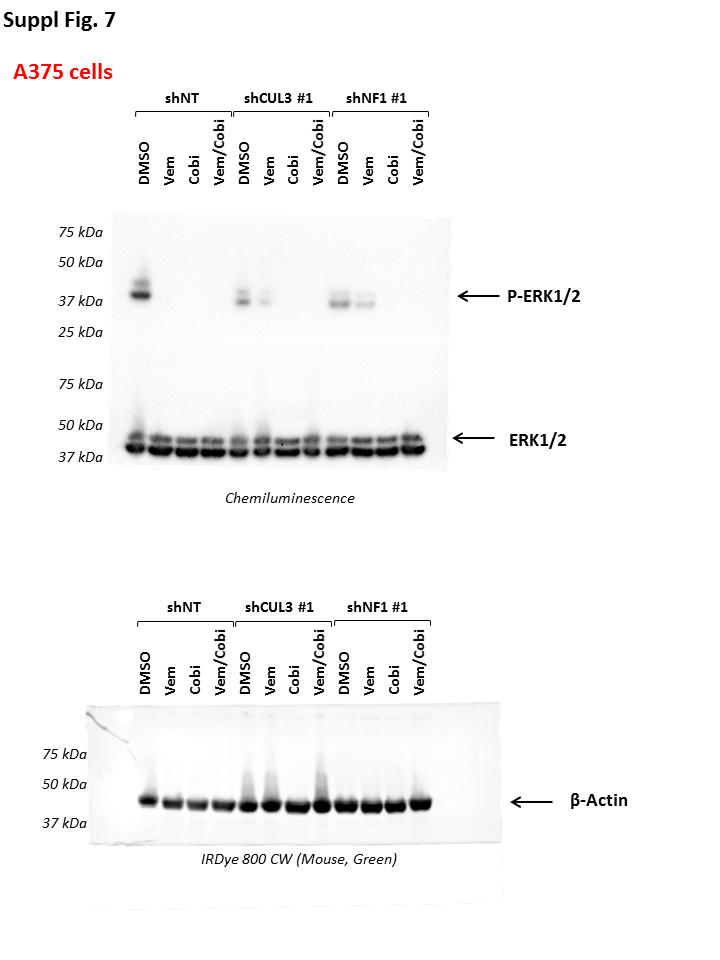
**

**
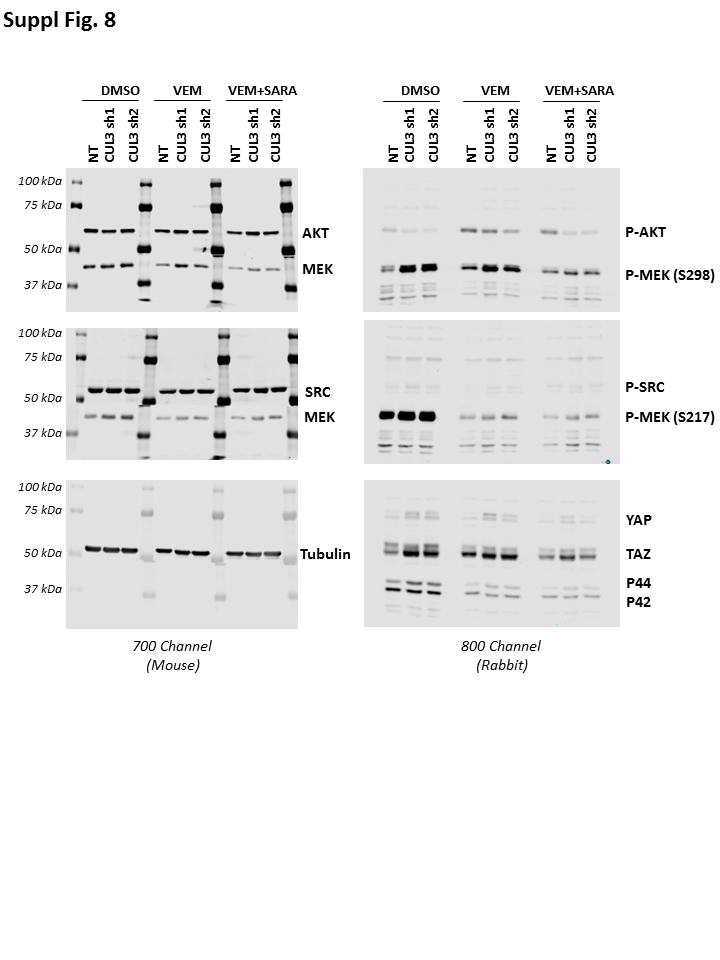
**

**
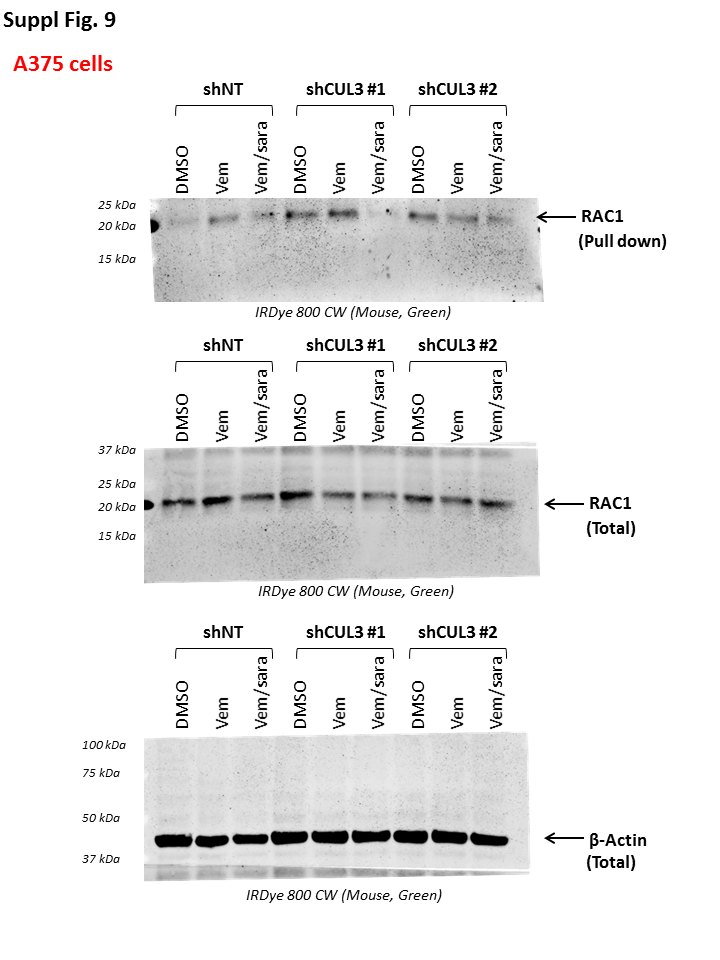
**

**
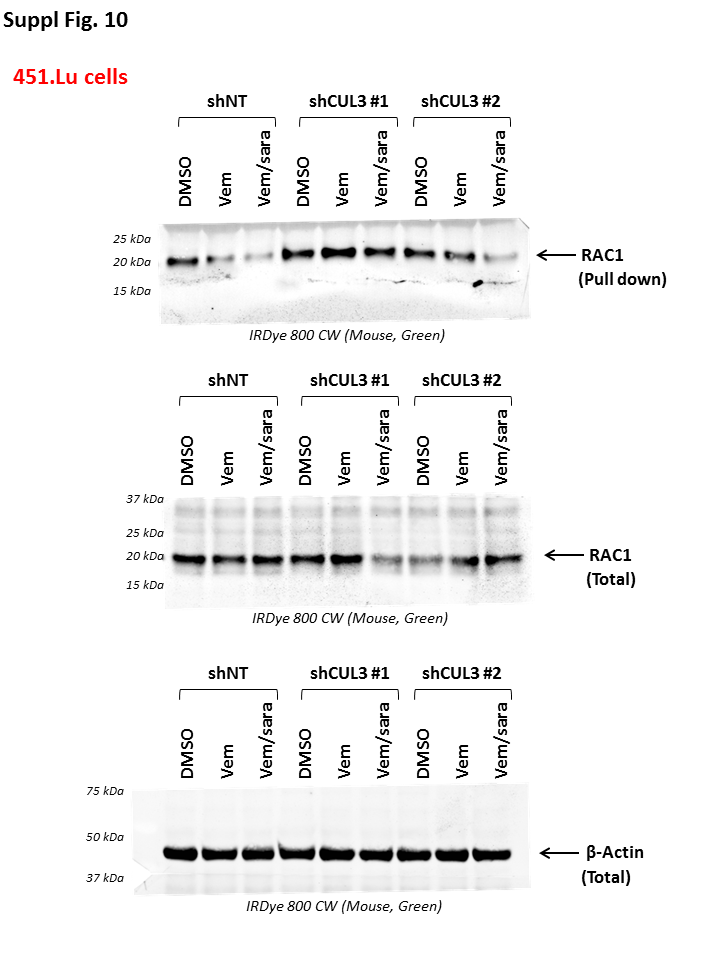
**

**
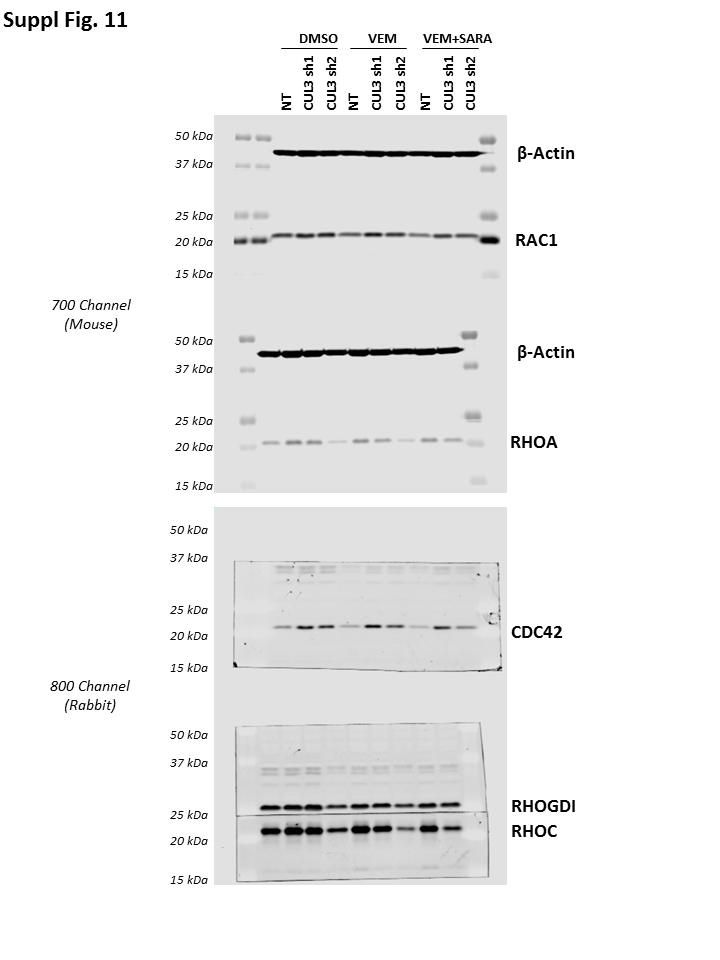
**

**
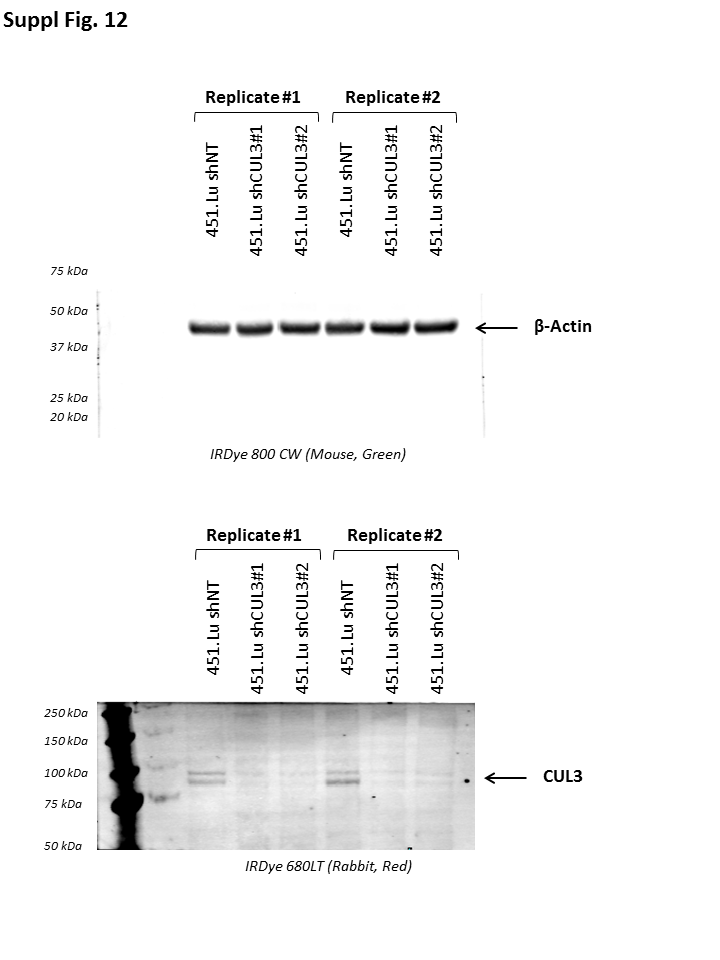
**

**
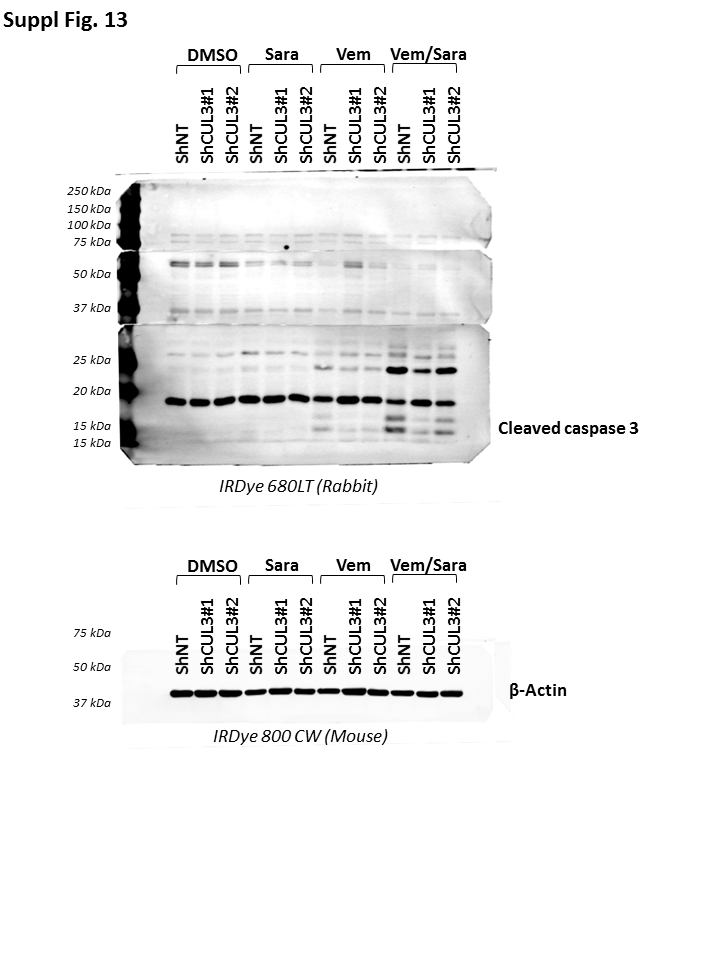
**

**
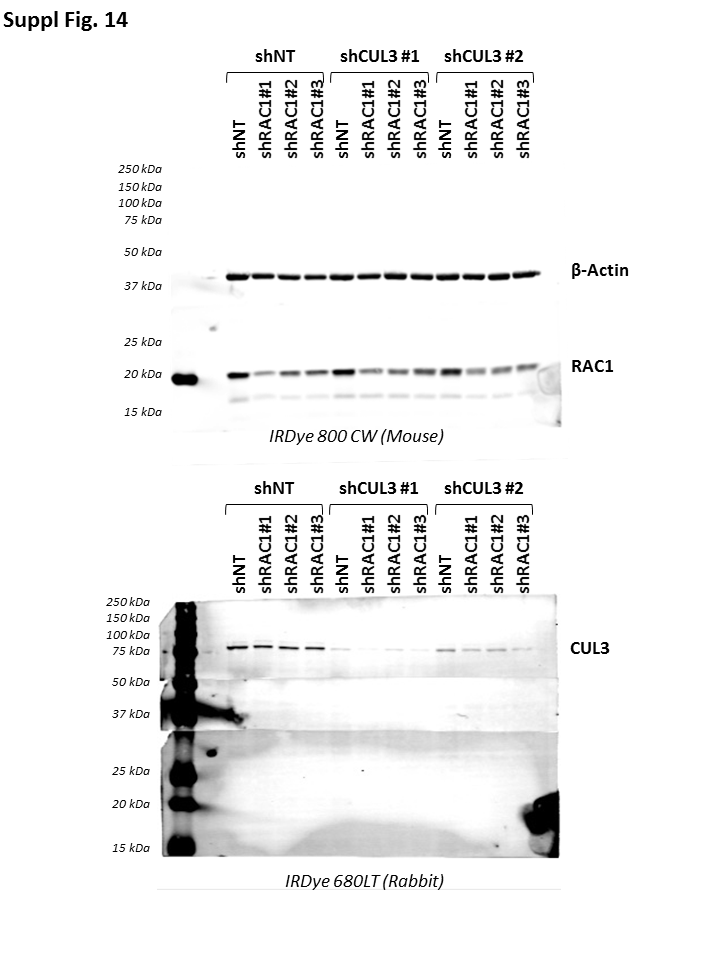
**
